# User Engagement, Demographics, and Health Status of the My ME-BYO Record, A Personal Health Management Mobile App: Retrospective Study

**DOI:** 10.2196/79109

**Published:** 2025-10-10

**Authors:** Choy-Lye Chei, Sho Nakamura, Kaname Watanabe, Hiroto Narimatsu

**Affiliations:** 1 Cancer Prevention and Control Division Kanagawa Cancer Center Research Institute Yokohama Japan; 2 Department of Genetic Medicine Kanagawa Cancer Center Yokohama Japan; 3 Graduate School of Health Innovation Kanagawa University of Human Services Kawasaki Japan; 4 Center for Innovation Policy Kanagawa University of Human Services Kawasaki Japan

**Keywords:** mobile health application, ME-BYO, user engagement, Japan, real-world data

## Abstract

We outlined the characteristics and health status of individuals using a mobile health app, based on a real-world user database.

## Introduction

**Figure 1:**
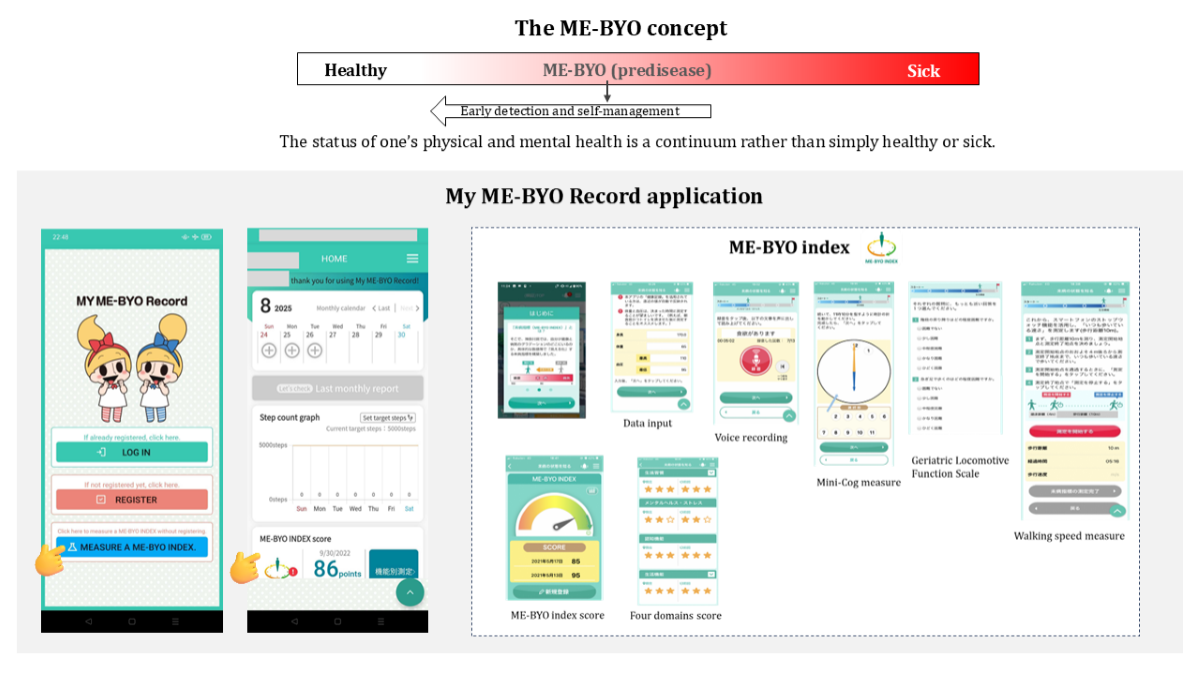
The ME-BYO concept, the My ME-BYO Record application, and the ME-BYO index.

The ME-BYO concept, proposed by the Kanagawa Prefecture in Japan, emphasizes the intermediate “predisease” state (ME-BYO) in health management (Figure 1) [1,2]. The My ME-BYO Record app, launched in 2017, records health and medication data and tracks physical activity. In 2020, the ME-BYO index was integrated to promote behavioral change by visualizing ME-BYO status (Figure 1) [2]. Unlike many Japanese mobile health (mHealth) apps from private companies, this app is developed and managed by the local government. Most research on mHealth app use relies on surveys [3], as developers rarely share app-collected data. We aimed to analyze user characteristics and health status using real-world data from the My ME-BYO Record app, with findings that may inform strategies to improve user engagement.

## Methods

### Overview

This study reports selected findings from a project commissioned by the Kanagawa Prefectural Government. Between April 1, 2020, and July 31, 2023, a cumulative total of 186,972 individuals registered as users; 10,239 aged ≥18 years who accessed the ME-BYO index at least once were included in the analysis.

The ME-BYO index is a self-reported 15-item assessment of metabolic, locomotor, cognitive, and mental resilience domains. Scores range from 0 to 100, with higher scores indicating better health [2] (Multimedia Appendix 1).

Differences between 1-time and repeat users were tested using *t* tests (continuous variables) and chi-square tests (categorical variables). Logistic regression examined the association between engagement (1-time users as reference) and independent variables (sex, age, and domain scores). Analyses were performed using Stata 17 SE (StataCorp). Further details are provided in Multimedia Appendix 1.

### Ethical Considerations

This study was approved by the Institutional Review Board of the Kanagawa Cancer Center (2024Eki-108). Informed consent was waived as only anonymous data were analyzed. No compensation was offered. The My ME-BYO Record app is freely available, and users consent to the Kanagawa Prefectural Government’s Terms of Use upon registration [4].

## Results

Of 10,239 users, 21.5% (n=2202) were repeat and 78.5% (n=8037) were 1-time users. Repeat users had significantly lower ME-BYO scores (Table 1). Logistic regression showed they were older, had lower cognitive function, and higher locomotor function than 1-time users (Table S1 in Multimedia Appendix 1).

**Table 1:** Characteristics of My ME-BYO Record users (N=10,239) from April 2020 to July 2023, stratified by 1-time and repeat users.

Characteristic		ME-BYO index users			*P* value^a^
		1-time users (n=8037)	Repeat users (n=2202)		
**Sex, n (%)**					<.001
	Women	3966 (49.3)	992 (45)		
	Men	4071 (50.7)	1210 (55)		
Age (years), mean (SD)		52.4 (12.8)	56.2 (11.7)	<.001	
**Age group (years), n (%)**					<.001
	18-29	441 (5.5)	56 (2.5)		
	30-39	921 (11.5)	138 (6.3)		
	40-49	1625 (20.2)	361 (16.4)		
	50-59	2596 (32.3)	744 (33.8)		
	60-69	1805 (22.5)	636 (28.9)		
	70-79	575 (7.2)	245 (11.1)		
	≥80	74 (0.9)	22 (1)		
ME-BYO score, mean (SD)		84.1 (10)	83.6 (10.1)	.02	
Metabolic function, mean (SD)		89.5 (11.4)	89.1 (11.4)	.13	
Locomotor function, mean (SD)		83.6 (21.1)	85.5 (19.8)	<.001	
Cognitive function, mean (SD)		84.3 (23.3)	80.8 (25)	<.001	
Mental resilience, mean (SD)		63.4 (21.4)	63.5 (21.5)	.97	

^a^*P* values were calculated using the *t* test for continuous variables and the chi-square test for categorical variables.

## Discussion

### Principal Findings

Approximately 20% (n=2202) of users were repeat users, indicating repeat engagement with the ME-BYO index for health monitoring was low. Although engagement may increase over time, the Prefectural Government may struggle to incorporate the app into regional health strategies until such growth occurs.

Repeat users were older than 1-time users. Users of mHealth apps supporting self-care and chronic disease monitoring also tend to be older than nonusers [5]. Older individuals may view the free ME-BYO index feature as a useful tool for monitoring well-being, leading to repeat engagement [6,7].

Repeat users also had lower ME-BYO index scores, particularly in cognitive function. As the app displays the latest ME-BYO index score on the home screen, repeat users may be motivated by disappointment with low scores and continue using the app to improve them [6]. Conversely, 1-time users might worry that subsequent scores would be worse. Lower cognitive ability has been associated with reduced mHealth access [8], yet individuals with chronic conditions are more likely to use mHealth apps than those without [9]. Our findings suggest that this app may help manage cognitive function in those with poorer cognitive abilities.

Repeat users also had higher locomotor function scores. Physically active individuals are more likely to use mHealth apps to manage health behaviors [9]. Similarly, those with better mobility may perceive the app as more useful for health monitoring, encouraging active engagement [7].

### Limitations

First, most app users were aged older than 40 years, limiting the generalizability of our findings to younger age groups. Second, there is potential for reporting bias through self-report. Although the Mini-Cog and walking speed measures in the ME-BYO index have been validated [10,11], other components still require validation. Third, the dataset lacked details such as disease comorbidities and socioeconomic status, which may affect user engagement. Including these confounders in future studies will improve the interpretation of the findings.

### Conclusions

This study highlights a gap between 1-time and repeat engagement with the ME-BYO index for health monitoring. Using a real-world database, our findings may help inform strategies to improve engagement.

## Appendix

Multimedia Appendix 1 Additional materials on the ME-BYO index, data analysis, and Table S1.

## Abbreviations

mHealth: mobile health

## Data Availability

In accordance with the Act on the Protection of Personal Information, public disclosure of the data is restricted. For details regarding personal data protection and the use of anonymized data, please refer to the Kanagawa Prefectural website.
