# Supplementary material for: User Engagement, Demographics, and Health Status of the My ME-BYO Record, A Personal Health Management Mobile App: Retrospective Study
Source: J Med Internet Res. 2025 Oct 10;27:e79109. doi: 10.2196/79109 (PMC12552808; doi:10.2196/79109)
Supplement: Multimedia Appendix 1 [file jmir_v27i1e79109_app1.docx]

**Multimedia Appendix 1.**

**Methods**

The ME-BYO index

The ME-BYO index comprises a self-reported 15-item assessment across four domains: metabolic function, locomotive function, cognitive function, and mental resilience. Users assessed their ME-BYO status by self-reporting age, sex, height, weight, systolic and diastolic blood pressure, responding to a three-question version of the Mini-Cog, completing a five-question Geriatric Locomotive Function Scale (GLFS-5), measuring walking speed and recording their voices using the app [1]. The app uses age, sex, body mass index, and blood pressure to assess metabolic function, the Mini-Cog to evaluate cognitive function, and the GLFS-5 plus walking speed to measure locomotor function [1]. The Mind Monitoring System (MIMOSYS; PST Inc., Yokohama, Japan) in the app analyzes recorded voice data to evaluate mental resilience [1]. After completing the assessment, the app displays the ME-BYO index score along with the individual score for each domain. Higher scores (ranging from 0 to 100) reflect better health status [1].

Data analysis

We categorized users into one-time users and repeat users. Mean values for age and ME-BYO index score, and percentages for sex and age groups were reported. Differences between one-time and repeat users were assessed using t-tests for continuous variables and chi-square tests for categorical variables. We examined the association between user engagement (with one-time users as the reference category) and independent variables (sex, age, and the individual domain scores) using a logistic regression model. Analyses were conducted using Stata 17 SE (StataCorp, USA), with statistical significance set at *P* < .05.

Reference

1. Nakamura S, Watanabe R, Saito Y, Watanabe K, Chung UI, Narimatsu H. The ME-BYO index: A development and validation project of a novel comprehensive health index. Front Public Health. 2023;11:1142281.

Table S1. Logistic regression analysis with the dependent variable: one-time versus repeat users (reference: one-time users).

| Independent variable | OR (95% CI)^a^ | *P* value^b^ |
| --- | --- | --- |
| Age, years | 1.025 (1.021-1.030) | <.001 |
| Sex (man=1) | 1.040 (0.943-1.147) | .43 |
| Metabolic function | 0.999 (0.994-1.003) | .52 |
| Locomotor function | 1.006 (1.004-1.008) | <.001 |
| Cognitive function | 0.996 (0.994-0.998) | <.001 |
| Mental resilience | 1.000 (0.998-1.002) | .83 |

^a^OR (95%CI): Odd ratio (95% confidence interval).

^b^*P* values were obtained from multivariable logistic regression.
